# Action of Calomel upon the Bile and as an Intestinal Disinfectant

**Published:** 1888-08

**Authors:** 


Action of Calomel upon the Bile and as an Intestinal Disinfectant.—J. Sawadsky has proved (*Wratsch.*, 1887, p. 17,) that calomel possesses a disinfectant action, which results from its transformation into an oxide of mercury under the influence of the alkalies of the bile in the intestine. The coloration of the stools after the ingestion of calomel should be attributed to the biliverdin in excess, which is liberated during this phenomenon of oxidation. This biliverdin is not altered on account of the antiseptic action of the oxide of mercury. These are the reasons why, after the administration of calomel, the reactions of this salt cannot be obtained from the intestinal contents.—*Gazette Hebdomadaire, March 2, 1888.*

